# Numerical Modeling of RC Beams Strengthened with Non-Pretensioned and Pretensioned NSM CFRP Strips

**DOI:** 10.3390/ma19112357

**Published:** 2026-06-02

**Authors:** Szymon Seręga, Renata Kotynia

**Affiliations:** 1 Faculty of Civil Engineering, Cracow University of Technology, 31-155 Kraków, Poland; 2 Faculty of Civil Engineering, Architecture and Environmental Engineering, Lodz University of Technology, 93-590 Łódź, Poland; renata.kotynia@p.lodz.pl

**Keywords:** strengthening, near-surface-mounted FRP, carbon fibre-reinforced polymer, prestressing, numerical modeling

## Abstract

This paper presents research on reinforced concrete beams strengthened with non-pretensioned and pretensioned near-surface-mounted (NSM) carbon fibre-reinforced polymer (CFRP) strips under self-weight and external preloading. The first part of this paper briefly describes and discusses the results of experimental tests performed on six beams with different reinforcing steel ratios, preloading levels, and strengthening-system configurations. Next, three-dimensional (3D) numerical models of the tested specimens were developed. The models consider the nonlinear behavior of concrete (both in tension and compression), steel bars, and the interface between concrete and CFRP laminates. For these models, the material parameters were established based on experiments and recommendations from the literature. Furthermore, a sensitivity analysis was conducted with respect to the material parameters of the model that were not directly obtained from experimental measurements. The analyses validated the applicability of the numerical model in predicting the flexural behavior of reinforced concrete (RC) members strengthened with near-surface-mounted (NSM) CFRP materials over the full loading range. Furthermore, the developed models were employed to assess the effectiveness of active strengthening relative to passive strengthening methods (i.e., without pretensioning of the laminate). A comparison study of actively and passively strengthened elements indicates that prestressing does not affect the ultimate limit state but enhances serviceability limit states. The presented computational model, together with the adopted computational strategy, demonstrates its effectiveness for analyzing realistic scenarios involving RC beams that are damaged and subjected to loading during the strengthening process.

## 1. Introduction

Among the numerous techniques available for strengthening reinforced concrete (RC) structures with fibre-reinforced polymer (FRP) laminates are two predominant approaches: the externally bonded (EB) method, in which the laminate is adhesively bonded to the surface of the structural element, and the near-surface-mounted (NSM) method, in which the laminate is embedded within a groove cut into the concrete cover [1]. The EB technique of strengthening structural elements is susceptible to damage from collisions, severe environmental conditions such as ultraviolet radiation, absorption of moisture, and elevated and high temperatures caused, for example, by fire. Moreover, a dominant failure mode of EB-type strengthening is governed by debonding between the laminate and the concrete. This results in the laminate not being fully utilized. To mitigate these disadvantages, the NSM technique was introduced into civil engineering practice over twenty-five years ago [1,2,3,4,5,6,7,8,9,10,11,12,13,14,15]. The high efficiency of this technique has been confirmed in many research campaigns [16,17,18,19,20,21,22,23,24,25,26,27,28,29,30]. The main reasons for the application of the NSM technique in flexural strengthening are as follows: better bond compared to externally bonded FRP reinforcement; limited damage by peeling-off failure due to flexural cracks; better mechanical response of the structure under serviceability loads; extended ductility of strengthened members; higher fatigue resistance; easier anchorage to prevent unexpected debonding in the maximum bending moment region of beams and columns in rigidly jointed frames; protection from mechanical damage, accidental impact, and vandalism, especially for the strengthening of negative bending moment regions of multiple-span members (beams and slabs); and the unchanged aesthetics of structures [1]. Despite the many advantages of NSM technology, it is not without its drawbacks [1]. First, the strengthened structure must be characterized by a sufficient thickness of concrete cover to allow for cutting a groove and embedding the laminate. In the case of insufficient cover, an additional overlay using special mortars is required. Applying the additional mortar layer is labor-intensive, cost-inefficient, and necessitates verification of the bond between the repair mortar and the existing concrete.

To increase strengthening efficiency, prestressing of FRP materials was introduced into practical applications. A review of the available literature on the flexural strengthening of RC members with pretensioned (active) NSM FRP strips or bars confirms the superior efficiency of this technique compared to passive strengthening techniques. The primary effect of pretensioning lies in the enhanced utilization of the tensile strength of FRP materials, which contributes to improved structural performance at the serviceability limit state [2,3,4,5]. Increasing the pretensioning level significantly increases the cracking loads and yielding loads [6,10,11], but it may not affect the ultimate loads for specimens with a tensile failure mode [12]. The most effective prestressing level of NSM CFRP strips was determined to range between 20% and 30% of the tensile strength of the laminate [12]. There are many other advantages of pretensioning introduced to CFRP laminates: reduced dead-load deflections; reduced crack and tensile steel reinforcement stress [14]; better bond behavior under monotonic and cyclic loading [15]; reduction in the risk of failure under fatigue loads [31,32,33,34,35]; and efficiency under environmental exposure [36,37]. However, strengthening using prestressed laminates decreases the ductility of the structure, which is undesirable from a structural safety perspective [38,39].

The results of tests confirmed excellent bond behavior between the rectangular CFRP rods and concrete cover, even without mechanical anchorage devices [7,8,9]. Moreover, the results reported in [9] showed that the prestress level applied to the CFRP laminate had a negligible influence on the prestress transfer length. This constitutes a major advantage over EB strengthening techniques. The effect of bonding conditions and CFRP anchorage systems was also studied in [40,41].

Numerical simulations employing the finite element method (FEM) constitute a significant tool for supporting experimental research campaigns. Because experimental studies are both time-consuming and expensive, FEM-based tools are increasingly becoming an indispensable alternative to laboratory tests. Moreover, a computational model offers supplementary information regarding the local and global behavior of structures (i.e., local slips of the embedded reinforcement or laminate and the tension-stiffening effect between adjacent cracks), which is often challenging to measure experimentally.

A comprehensive literature review demonstrates that the finite element method has been used in the analysis of concrete structures strengthened with FRP composites using the NSM technique. Typically, numerical models developed based on experimental data are employed to extrapolate the analysis to scenarios involving parameters not explicitly examined during experimental tests. Numerical simulations have successfully replicated the behavior of both reinforced concrete and prestressed concrete beams strengthened with pretensioned CFRP NSM composites, as described in [40,41,42,43,44]. In [40], the strength of eight full-scale RC beams was tested and then modeled in ABAQUS version 6.71 using the CDP model for concrete and a spring element for the bond–slip interface between laminate and concrete. The finite element model predicted cracking, yielding, and an ultimate load with a maximum error of 6%. A parametric study showed that each 12% increase in laminate length improved structural performance by approximately 5% across all load levels. Moreover, each 10% increase in prestressing force increased the cracking load by more than 13%, while the effect on the ultimate load was modest. Similarly, in the numerical study reported in [41], the ABAQUS finite element code was used in the numerical simulations with the same material models as in [40]. A 3D model was developed to study the effect of bond length and prestressing level on the load-displacement behavior of RC beams. The proposed model predicted all load levels with errors below 5%. The parametric study demonstrated that a fourfold increase in the prestressing force resulted in approximately 26% and 18% increases in the cracking load and ultimate load, respectively. Furthermore, a 28% increase in the laminate length resulted in a 13% increase in the cracking load and a 10% increase in the ultimate load. The primary objective of the study in [42] was to determine the optimal prestressing level of the laminate from the perspective of structural ductility. For this purpose, a 3D finite element model was developed in ANSYS version 12. Concrete was modeled using a multilinear plasticity model with the Willam–Warnke yield criterion, whereas the concrete-to-laminate behavior was described by a mixed-mode cohesive zone model. Using the criterion that the energy absorption of the strengthened beam must equal that of the unstrengthened specimen, the numerical model identified an optimum prestressing level of about 30%. The authors of [43] investigated the flexural behavior of reinforced concrete beams strengthened with passive and prestressed NSM CFRP bars through combined experimental testing and nonlinear finite element analysis in ANSYS. A 3D model was proposed with the same assumptions as in [42]. Following experimental validation (mean error of ~2.58%), an extensive parametric study was carried out, examining the influence of CFRP bar length, tensile steel corrosion, concrete compressive strength, NSM reinforcement prestress level, and the axial stiffness of the prestressed NSM joint. It was demonstrated that the ultimate load capacity increased with CFRP bar length up to a threshold of 175 bar diameters (beyond this length, further extension yielded negligible benefit); progressive steel corrosion and reduced bar length were found to significantly diminish strengthening efficiency; higher concrete compressive strength delayed the onset of concrete cover separation. Moreover, prestressing the NSM reinforcement effectively enhanced cracking and yielding loads. However, prestressing did not appreciably improve the ultimate load due to premature rupture of the laminates. The authors of [44] discussed the mechanical behavior of prestressed concrete girders strengthened with pretensioned CFRP laminates. The girders were modeled in 2D using ANSYS with a linear-elastic model for concrete with a perfect bond between the concrete and laminate. Although the numerical model proposed in [44] was not capable of accurately reproducing the cracking stage, steel yielding, and ultimate load capacity, the following conclusions were formulated: post-tensioned NSM CFRP effectively reduced steel strain, improved serviceability, increased upward camber, and enhanced the flexural functionality of the girders under service loads.

Advanced two- and three-dimensional simulations have also been used to replicate crack patterns and failure mechanisms in beams strengthened using non-pretensioned CFRP laminates [45,46,47,48,49,50,51].

In [45], a 3D model was developed in ANSYS using a multilinear plasticity model with the Willam–Warnke yield criterion for concrete. The interfaces between the groove filler and the surrounding concrete, as well as between the FRP laminate and the groove filler, were modeled separately. The model successfully reproduced the observed failure modes, including laminate pull-out, concrete cover delamination, and flexural crushing. Numerical studies revealed that CFRP rods surpass AFRP (aramid) and GFRP (glass) in ultimate strength by 18.5% and 43.8%, respectively, and that increasing the rod diameter from 6 to 16 mm raises the failure load by 83.6%. The same computational tool was used in [46] to develop a three-dimensional model with elastic-plastic behavior assumed for concrete and a perfect bond assumption at all interfaces. The numerical results agreed well with the experimental load-deflection curves, crack patterns, and strain distributions along the GFRP bars, particularly before the yielding of steel. The numerical simulations revealed that the absence of an end anchorage had a marginal effect on the load capacity, while the confinement conditions around the bent end anchorages were decisive. In [47], the authors proposed a 2D nonlinear model in MSC.MARC finite element code to predict end-cover separation failures in RC beams strengthened with either EB or NSM FRP. Concrete was modeled using an orthogonal fixed smeared-crack model. Bond–slip relationships at all interfaces were considered. Numerical validation demonstrated that including the radial stress effect provided by tensile steel bars is a crucial parameter for modeling the cover separation. Concrete cover separation failure mode was also investigated in [48]. A 3D nonlinear FE model was developed in ABAQUS to simulate RC beams strengthened with novel T-shaped CFRP profiles combining NSM and EB techniques. Concrete nonlinearity was captured via the Concrete Damaged Plasticity model. Two surface-based contact interfaces at the CFRP–epoxy and concrete–epoxy boundaries were governed by mixed-mode damage laws with linear softening. The model accurately reproduced flexural responses and identified intermediate crack debonding with concrete cover splitting as the governing failure mode. Additional numerical simulations showed that a higher CFRP elastic modulus increased post-yield stiffness without altering the failure mechanism, and lower concrete strength reduced resistance to cover separation. Also, it was indicated that a larger ratio of bond contact area to cross-sectional area of the CFRP profile delayed premature failure. Advanced numerical modeling was also employed in [49], where the ATENA software (version 5.6) was utilized for computational analysis. A 3D FE model of RC beams retrofitted with NSM CFRP textile and laminates using epoxy and cement-based adhesives was developed. Concrete and mortar were modeled with a fracture-plastic constitutive law combining exponential tension softening and the Rankine failure criterion, with crack opening governed by a fictitious crack model based on fracture energy. The model incorporated nonlinear bond–slip relationships between the concrete and the laminate. The results showed good agreement with the experimental load-deflection curves, crack patterns, and strain distributions for both epoxy and cement-based adhesives. In [50], a 2D nonlinear FE model was developed in ABAQUS to study the effect of load uniformity on debonding in RC beams strengthened with NSM CFRP strips. Concrete cracking was simulated using the crack band model based on the smeared-crack concept. Nonlinear bond–slip relationships at the steel–concrete interface and laminate–concrete interface were considered. In the case of the steel–concrete interface, the radial stresses due to slips of deformed bars were taken into account. It was demonstrated that the beneficial effect of higher load uniformity on debonding resistance decreased with increasing NSM FRP bond length. In [51], the influence of the axial stiffness of the laminate and the confinement of the concrete cover on the behavior of strengthened reinforced concrete beams was investigated using a 3D finite element model in ABAQUS. Concrete was simulated using the Concrete Damaged Plasticity model. A perfect bond between the concrete and laminate was assumed. Model validation against six experimental beams demonstrated good agreement in load-deflection response and failure modes, with the highest discrepancy observed for concrete cover separation failures. The study showed that beyond a critical stiffness ratio of 1.25, failure consistently transitioned to concrete cover separation, indicating the necessity of end anchorage systems above this threshold.

Besides the simulation of the global response of strengthened flexural structures, nonlinear computations have been used in the analysis of local effects, i.e., the bond–slip or normal traction-separation mechanisms between the CFRP laminate in the groove and concrete (see, for example, [52,53,54,55,56,57]).

The review of the current research summarized above indicates that due to the very significant and multivariate material nonlinearities, FEM analysis is not often used to simulate and reproduce experimental tests. Furthermore, none of the numerical simulations described in the literature consider the fact that in practical applications, strengthening systems are usually applied to structural elements under load. Frequently, structural elements are seriously damaged (cracked) before strengthening, and the NSM CFRP strengthening system is often applied to elements in which such cracks are not repaired. In light of the above considerations, the aim of this paper is to present a computational procedure based on FEM for reinforced concrete elements strengthened with pretensioned or partly pretensioned NSM CFRP laminates applied to damaged (cracked) flexural elements that remain under self-weight or self-weight and an external load during strengthening. This paper attempts to fill this research gap by proposing a computational procedure and an FEM-based computational model using commercially available software [58], developed based on the experiment reported in [59] and briefly summarized in Section 2. The presented calculation procedure can be used in simulations extending the range of studied elements. Furthermore, the proposed computational procedure can be employed in expert-oriented design problems concerning the strengthening of reinforced concrete structures in cases where complete unloading prior to strengthening is not possible.

## 2. Experimental Database

### 2.1. Summary of the Experimental Program and Procedure

**Figure 1:**
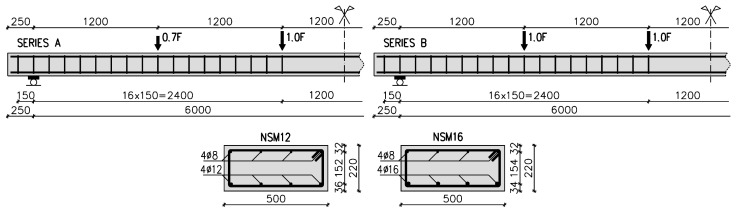
Geometry of tested specimens, static schemes, and details of steel reinforcement.

Six beams with rectangular cross-sections measuring 500 mm in width and 220 mm in height were experimentally investigated under a six-point bending loading scheme. The total span length of each beam was 6.0 m. The load was applied monotonically using two hydraulic jacks with a maximum capacity of 100 kN. The force from each actuator was transferred to the RC beams through a rigid steel spreader beam supported at two points on the slab. The configuration of the loading points on the beams is shown in Figure 1. The beams were categorized into two series: A and B, depending on the quantity of NSM CFRP strips employed. The following designations were used to describe the beam nomenclature: NSM12 and NSM16 mean beams reinforced with longitudinal steel bars of a diameter of 12 and 16 mm, respectively; A and B refer to Series A and Series B. Series A comprised two beams (NSM12A and NSM16A) strengthened with a combination of one pretensioned and two passive CFRP strips. Series B consisted of four specimens (NSM12B, NSM16B, NSM12B-L, and NSM16B-L), all strengthened with two pretensioned CFRP strips; L refers to the external preloading applied to the beam before strengthening. The bottom tensile steel reinforcement consisted of four bars with a nominal diameter of 12 mm, with a steel reinforcement ratio of $\rho_{s}=0.49\%$ for beams with the acronym NSM12, and four bars with a nominal diameter of 16 mm ($\rho_{s}=0.87\%$) for beams designated NSM16 (Figure 1).

The beams were strengthened under two distinct preloading levels. The first level corresponded to self-weight, which induced tensile stresses in the longitudinal steel reinforcement of 25% and 14% of the yield strength in the unstrengthened members for beams NSM12 and NSM16, respectively. The second preloading level corresponded to 60% of the steel yield strength in the unstrengthened beam. This loading level was achieved through the combined action of self-weight and external preloading. The corresponding values of the bending moment associated with preloading are summarized in Table 1.

The beams were fabricated using commercially available concrete of Class C50/60. The mean compressive and tensile strengths of concrete and the modulus of elasticity (*E_cm_*) are summarized in Table 2. The mechanical parameters of concrete were determined on the testing days through uniaxial compression tests conducted on 150 × 300 mm^2^ cylinders (*f_c_*) and 150 mm cube samples (*f_c,cube_*). The tensile strength (*f_ct,sp_*) was determined based on splitting tests on 150 mm cube samples. The mechanical parameters of steel bars (elastic modulus *E_s_*, yield stress *f_y,_* and tensile strength *f_u_*) were determined based on uniaxial tensile tests. The same testing procedure was employed to determine the tensile strength (*f_fu_*), elastic modulus (*E_f_*), and ultimate strain (*ε_fu_*) of the CFRP laminate. The results are presented in Table 2.

**Figure 2:**
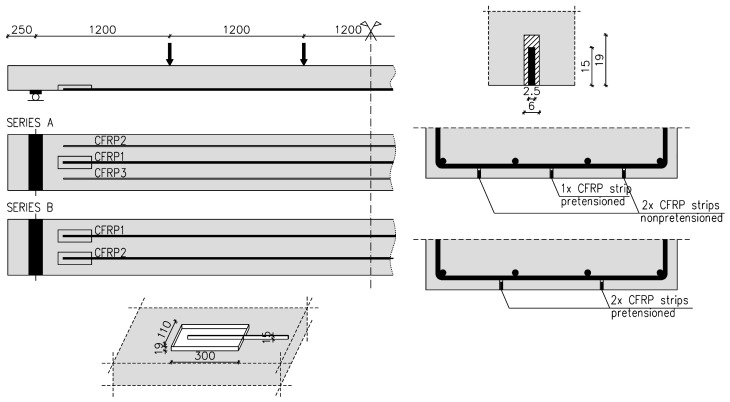
Configurations of CFRP strips.

**Figure 3:**
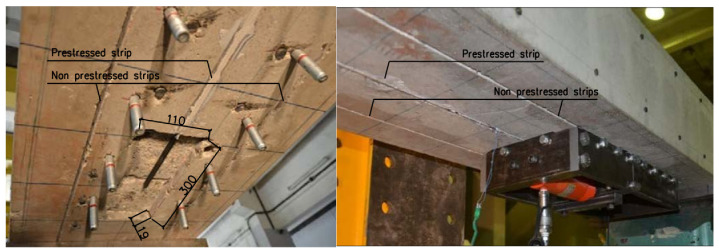
Strengthening system.

The beams were strengthened with CFRP strips 2.5 mm wide and 15 mm high, corresponding to composite reinforcement ratios of $\rho_{f}=0.1\%$ and $\rho_{f}=0.07\%$ for Series A and B, respectively. A two-component epoxy adhesive was applied to bond the CFRP strips within the grooves. The strengthening system was installed under self-weight (NSM12A, NSM16A, NSM12B, NSM16B) or under external preloading (NSM12B-L, NSM16B-L). In order to best reflect a real strengthening scenario, the CFRP strips were installed in the overhead position under the test stand. The widened rectangular gaps were 110 mm wide, 300 mm long, and 19 mm deep for mounting the pretension system (Figure 2 and Figure 3). The pretensioning force was applied to the CFRP strips under strain control to a level of approximately 6‰. This value corresponds to 33% of the ultimate tensile strain (rupture strain) of the CFRP strips. To introduce the pretensioning force into a narrow CFRP strip, patented prestressing equipment was used [61].

Beams belonging to Series A were strengthened with a single pretensioned CFRP strip. The central groove was filled with adhesive into which the CFRP strip was embedded and subsequently cured at 90 °C for 45 min. The remainder of the strip was not subject to accelerated curing. Upon curing of the anchoring adhesive, the pretensioning force decreased by approximately 50%. Following the pretensioning of the central CFRP strip (Figure 2), it was anchored to clamps installed within a slot formed in the concrete cover and maintained for an additional 12 h prior to full release. Two passive CFRP strips were subsequently bonded into the remaining lateral grooves. The combination of active and passive strengthening was used due to space limitations between the NSM pretensioning system and the two lateral strips (Figure 3). In the case of Series B, a sequential prestressing procedure was applied. Upon completion of the prestressing and bonding procedure for the first strip, the system was relocated to the second strip, and the prestressing/anchoring procedure was repeated. Following the completion of the strengthening procedure, the element was subjected to monotonic loading up to failure.

The following set of mechanical quantities was measured throughout the experiments: vertical displacements at the midspan and near the loading points using linear displacement transducers, and strains along the laminate at a spacing of 600 mm. Moreover, concrete strains were recorded in the tensile and compressive zones. These strains were averaged over a 300 mm long gauge length.

### 2.2. Summary of Test Results

**Figure 4:**
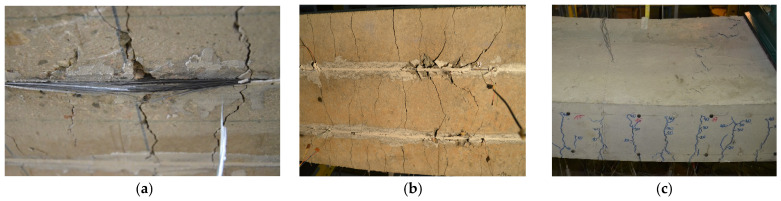
Beam failure: (**a**) NSM12A beam—rupture of pretensioned strip; (**b**) NSM12B-L beam—rupture of pretensioned strips; (**c**) NSM16A beam—concrete crushing.

All beams exhibited flexural failure attributed to the rupture of both the active and passive CFRP strips. In the case of Series A, the pretensioned strip ruptured prior to the passive strip (Figure 4). This failure mode confirmed the full utilization of the tensile strength of both the pretensioned and passive CFRP strips. Subsequent to the rupture of the CFRP strips, concrete crushing in the compression zone was observed in beam NSM16A (Figure 4c).

**Figure 5:**
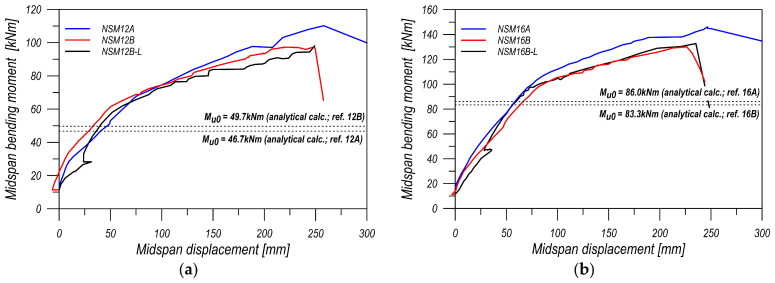
Experimental results. Midspan bending moment versus midspan deflection: (**a**) NSM12-type specimens; (**b**) NSM16-type specimens.

The key mechanical quantities measured during the experiments are summarized in Figure 5 and Table 3. Figure 5 compares the load-displacement paths of the tested elements and presents the predicted ultimate load-bearing capacities of the reference specimens (unstrengthened specimens not tested experimentally) based on simplified cross-sectional analysis. The results of the tests confirmed the strong influence of the tensile steel reinforcement ratio on the strengthening efficiency. Beams with a lower steel reinforcement ratio (NSM12) exhibited a much higher increase in the load-bearing capacity than beams with a higher reinforcement ratio (NSM16) (compare Figure 5 and Table 3). Moreover, it was confirmed that external preloading had an insignificant influence on the load-bearing capacity.

Pretensioning of the CFRP strips resulted in negative (reverse) vertical displacements that appeared after the pretensioning process (Table 3). The magnitude of these displacements depended on the preloading and on the steel reinforcement ratio. Generally, the higher the level of preloading, the larger the negative displacements observed during the prestressing of CFRP laminates. Similarly, a higher degree of steel reinforcement resulted in smaller negative (reverse) displacements. A more detailed discussion on the experimental results can be found in [59].

## 3. Numerical Analysis

### 3.1. Finite Element Model

**Figure 6:**
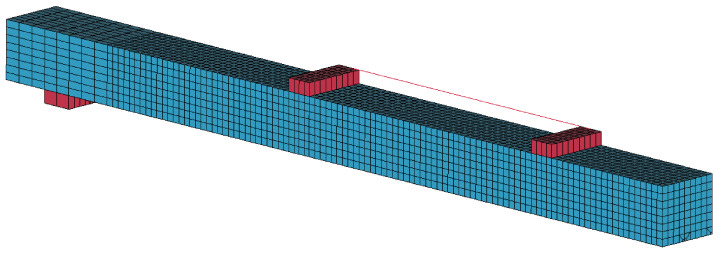
Topology of the finite element mesh of partially prestressed beams (Series A).

**Figure 7:**
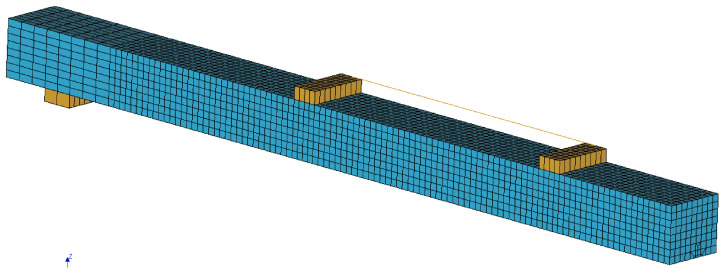
Topology of the finite element mesh of fully prestressed beams (Series B).

The numerical analysis was carried out using the DIANA FEA version 10.6 software [58]. A three-dimensional model of the experimentally tested beams was developed, exploiting the double symmetry of the geometry, boundary conditions, and loading configuration. The finite element mesh topologies adopted for the analyses are presented in Figure 6 and Figure 7 for the beams with partial (Series A) and full prestressing (Series B), respectively. The finite element mesh of the concrete matrix was composed of 20-node hexahedral brick elements (CHX60) with a maximum edge dimension of 28 mm. The selection of the finite element size and the analysis of the effect of mesh discretization on the computational results are discussed in Section 3.4. Additional components, including steel plates for load transmission and support, were also incorporated into the model. The contact interface between the steel load-transfer plates and the beam surface was modeled using eight-node zero-thickness interface elements (CQ48I).

**Figure 8:**
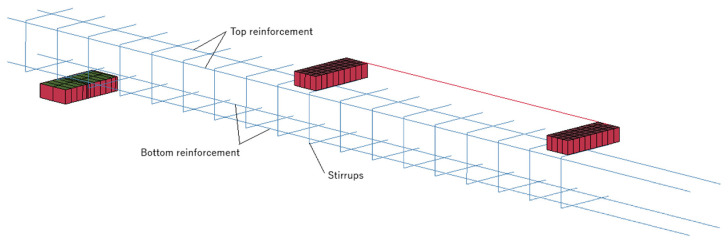
Layout of steel reinforcement.

The bottom reinforcing bars were modeled using two-node truss elements (L2TRU). The external dimensions and the reinforcement layout were consistent with those of the experimental program described in Section 2 and are shown in Figure 8. The reinforcing bars were connected to the concrete matrix via interface elements. The interface formulation for the reinforcement-to-concrete connection is capable of modeling relative displacements (slips) between the concrete matrix and the reinforcing bars in the direction tangential to the reinforcement, independently of the topology of the concrete matrix elements. Consequently, the nodes of the steel bars are not required to coincide with the nodes of the three-dimensional elements. The displacements of the concrete matrix along the bar are evaluated through interpolation of the displacement field of the corresponding three-dimensional element. In the case of the stirrups and the top reinforcement, local slips in the vicinity of cracks do not exert a significant influence on the overall behavior of the beams. Accordingly, the fully embedded reinforcement concept was adopted to represent this type of reinforcement.

**Figure 9:**
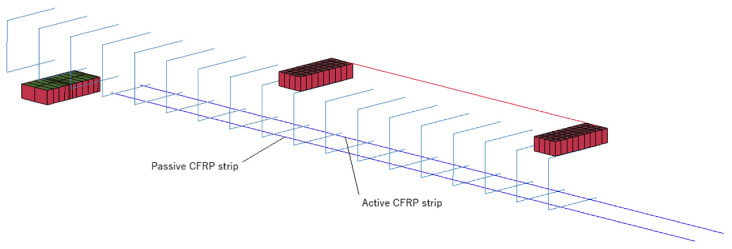
Layout of CFRP laminate of partially prestressed beams (Series A).

**Figure 10:**
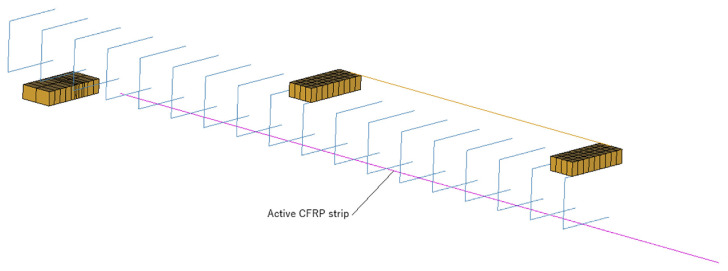
Layout of CFRP laminate of fully prestressed beams (Series B).

The CFRP laminates were modeled using a three-node beam element (CL18B), quadratically interpolated along the element axis (see Figure 9 and Figure 10 for Series A and Series B, respectively). The cross-section of these elements had a rectangular shape with dimensions of 2.5 mm × 15 mm. The normal stresses were numerically integrated over the cross-section in both directions. This enabled the incorporation of nonlinear stress–strain relationships for the CFRP strip modeled as a one-dimensional element. The slips between the concrete and the laminate were modeled using one-dimensional interface elements—CL18I with a slip perimeter of 32.5 mm.

### 3.2. Constitutive Models for Materials

#### 3.2.1. Concrete

**Figure 11:**
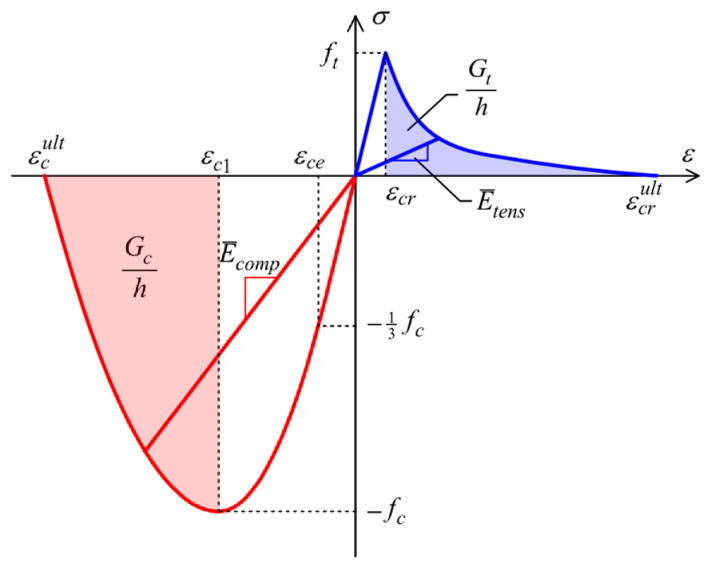
Uniaxial stress–strain relationships for concrete.

The constitutive model for concrete adopted in the analysis is based on the smeared-crack concept and is formulated in terms of total strains, following the approach presented in [62]. Since significant reorientation of damage is not expected in the modeled problem, the fixed-crack smeared-crack model was adopted. Prior to cracking, the secant stiffness is determined from the stress–strain relationships established in the directions of the principal strains. Upon initiation of the first crack, the local coordinate directions are fixed and remain coaxial with the crack plane. At each integration point, the stiffness values are determined from the stress–strain relationships expressed in the coordinate system defined by the first crack. Subsequent cracks may develop exclusively in directions perpendicular to the first crack. The constitutive relationship based on the secant stiffness matrix is expressed by the following formula [62]:

(1) $$\sigma =D_{sec}\varepsilon ,$$

where $\sigma ={\left[\begin{array}{cccccc} \sigma_{c1} & \sigma_{c2} & \sigma_{c3} & \tau_{c12} & \tau_{c23} & \tau_{c13} \end{array}\right]}^{T}$ represents the stress vector (T denotes matrix transposition), $\varepsilon ={\left[\begin{array}{cccccc} \varepsilon_{c1} & \varepsilon_{c2} & \varepsilon_{c3} & \gamma_{c12} & \gamma_{c23} & \gamma_{c13} \end{array}\right]}^{T}$ is the strain vector, and $D_{sec}$ is the secant stiffness matrix. In Equation (1), the instantaneous mechanical component of strain ε is decomposed from the total strains $\varepsilon_{tot}$ as follows: $\varepsilon =\varepsilon_{tot}-\varepsilon_{sh}$, where $\varepsilon_{sh}=-\left|\varepsilon_{cs}\left(t\right)\right|{\cdot \left[\begin{array}{cccccc} 1 & 1 & 1 & 0 & 0 & 0 \end{array}\right]}^{T}$ and $\varepsilon_{cs}\left(t\right)$ is the mean shrinkage strain due to cement hydration and concrete drying according to Eurocode 2 [63]. The secant stiffness matrix has a diagonal form

(2) $$D_{sec}=\left[\begin{array}{cc} D_{sec}^{\sigma } & 0\\ 0 & D_{sec}^{\tau } \end{array}\right],\;D_{sec}^{\sigma }=\left[\begin{array}{ccc} {\bar{E}}_{c1} & 0 & 0\\ 0 & {\bar{E}}_{c2} & 0\\ 0 & 0 & {\bar{E}}_{c3} \end{array}\right],D_{sec}^{\tau }=\left[\begin{array}{ccc} G_{c12} & 0 & 0\\ 0 & G_{c23} & 0\\ 0 & 0 & G_{c13} \end{array}\right]\;$$

where $G_{c12}={\bar{E}}_{c1}{\bar{E}}_{c2}{\left({\bar{E}}_{c1}+{\bar{E}}_{c2}\right)}^{-1}$, $G_{c23}={\bar{E}}_{c2}{\bar{E}}_{c3}{\left({\bar{E}}_{c2}+{\bar{E}}_{c3}\right)}^{-1}$, and $G_{c13}={\bar{E}}_{c1}{\bar{E}}_{c3}{\left({\bar{E}}_{c1}+{\bar{E}}_{c3}\right)}^{-1}$ are the shear moduli. In each direction, the normal stiffness values ${\bar{E}}_{c1}$, ${\bar{E}}_{c2}$, are ${\bar{E}}_{c3}$ are obtained from the uniaxial stress–strain relationship presented in Figure 11.

Upon cracking of the concrete, the shear modulus corresponding to the crack direction is multiplied by a constant shear retention factor β≪1.0. The secant stiffness matrix defined by Equation (2) contains only diagonal terms. Consequently, Equation (1) does not capture the Poisson effect, irrespective of whether the concrete is subjected to tension or compression. The lateral expansion strains arising from this effect are incorporated into the load vector in accordance with the procedure described in [62].

The uniaxial stress–strain relationship of concrete comprises two distinct parts. For the tensile regime, this relationship is defined as [64,65]

(3) $$\sigma_{t}=\left\{\begin{array}{cc} E_{c}\cdot \varepsilon & 0\leq \varepsilon \leq \varepsilon_{cr}\\ f_{t}\left(\begin{array}{c} \left(1+{\left(c_{1}\frac{\varepsilon -\varepsilon_{cr}}{\varepsilon_{cr}^{ult}}\right)}^{3}\right)\cdot \exp\left({-c}_{2}\frac{\varepsilon -\varepsilon_{cr}}{\varepsilon_{cr}^{ult}}\right)\ldots \\ \ldots -\frac{\varepsilon -\varepsilon_{cr}}{\varepsilon_{cr}^{ult}}\left(1+c_{1}^{3}\right)\cdot \exp\left(-c_{2}\right) \end{array}\right) & \varepsilon_{cr}<\varepsilon \leq \varepsilon_{cr}^{ult}\\ 0 & \varepsilon >\varepsilon_{cr}^{ult} \end{array}\right.$$

where $E_{c}$ is the concrete elastic modulus, $f_{t}$ is the concrete tensile strength, $\varepsilon_{cr}=f_{t}/E_{c},$ and constants $c_{1}=3.0$ and $c_{2}=6.93$ are taken from [65]. Mesh objectivity for concrete in tension is ensured through the preservation of the constant fracture energy $G_{t}$ [66]. In accordance with the constant-fracture-energy approach, the ultimate tensile strain $\varepsilon_{cr}^{ult}$ is calculated as $\varepsilon_{cr}^{ult}=\varepsilon_{cr}+5.136\cdot G_{t}\cdot h^{-1}\cdot f_{t}^{-1}$, where $h=\sqrt[{3}]{V_{FE}}$ is a crack bandwidth and $V_{FE}$ is the volume of a finite element [67]. The tensile strength and elastic modulus of concrete were adopted from the experimental program described in Section 2, Table 2. The fracture energy $G_{t}$ was estimated in accordance with the provisions of Model Code 2010 [68], based on the experimentally determined compressive strength reported in Table 2. The Poisson’s ratio was taken as 0.2 [63]. The modeled beams exhibited a failure mode characteristic of flexural behavior. Under these conditions, diagonal cracking induced by shear stresses was negligible. Consequently, the structural response was not governed by the shear behavior of concrete. Therefore, a constant shear retention factor of β=0.1 was adopted in the simulations, based on the recommendations in [69,70,71,72].

The formula for the uniaxial stress–strain curve for concrete in compression is described by Equation (5) [73]:

(4) $$\sigma_{c}=\left\{\begin{array}{cc} -\frac{1}{3}f_{c}\cdot \frac{\varepsilon }{\varepsilon_{ce}} & \varepsilon_{ce}<\varepsilon \leq 0\\ -\frac{1}{3}f_{c}\left(1+4\frac{\varepsilon -\varepsilon_{ce}}{\varepsilon_{c1}-\varepsilon_{ce}}{-2\left(\frac{\varepsilon -\varepsilon_{ce}}{\varepsilon_{c1}-\varepsilon_{ce}}\right)}^{2}\right) & \varepsilon_{c1}<\varepsilon \leq \varepsilon_{ce}\\ -f_{c}\left(1{-\left(\frac{\varepsilon -\varepsilon_{c1}}{\varepsilon_{c}^{ult}-\varepsilon_{c1}}\right)}^{2}\right) & \varepsilon_{cr}^{ult}<\varepsilon \leq \varepsilon_{c1}\\ 0 & \varepsilon \leq \varepsilon_{c}^{ult} \end{array}\right.$$

where $f_{c}$ is the compressive concrete strength, $\varepsilon_{ce}=-1/3{\cdot f}_{c}/E_{c}$, and $\varepsilon_{c1}=-5\varepsilon_{ce}$. The compressive stress–strain relationship of concrete is depicted in Figure 11. Experiments [74,75,76] carried out on concrete in a uniaxial stress state confirmed that, for the post-peak behavior under compression, crushing of concrete localizes to a certain zone. This implies that the descending branch of the stress–strain relationship is size-dependent and, consequently, a stress-displacement description is more appropriate in this context than a stress–strain formulation. In order to retain the stress–strain form of the material constitutive model while simultaneously ensuring the objectivity of the post-peak compressive behavior of concrete independently of the finite element mesh discretization, the strain $\varepsilon_{c}^{ult}$ in Equation (4) is evaluated as $\varepsilon_{c}^{ult}=\varepsilon_{c1}-1.5\cdot G_{c}/\left(h\cdot f_{c}\right)$, where $G_{c}$ denotes the compressive fracture energy and h is the characteristic length of a finite element, which is assumed to be identical to that adopted for tension. The $G_{c}$ value is an additional material property and is calculated from the post-peak stress-displacement diagram. The fracture energy in compression was assumed to be equal to $G_{c}=250G_{t}$, in accordance with the recommendation in [77]. The lateral effects of cracking and confining stresses on compressive behavior were assumed according to [62,78], respectively. If concrete is in an unloading stage, the stress–strain path follows a linear trajectory directed toward the origin (Figure 11), indicating that the material model does not account for plastic strains.

#### 3.2.2. Reinforcing Steel

The constitutive relationships for the bottom and top reinforcement were modeled as elastic-plastic with kinematic hardening. The mechanical parameters governing the uniaxial stress–strain behavior of steel were adopted from Table 2. The stirrups were modeled using a linear elastic model with the elastic modulus taken from Table 2.

#### 3.2.3. CFRP Laminate

**Figure 12:**
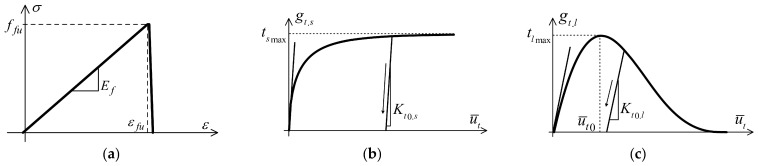
(**a**) Stress–strain relationship for laminate; (**b**) bond–slip law for steel-to-concrete interface; (**c**) bond–slip law for laminate-to-concrete interface.

A linear-elastic constitutive relationship for the CFRP laminate was assumed. For computational reasons, a short plateau (0.5‰) was introduced after reaching the tensile strength, followed by a linear stress drop to zero over the subsequent 0.5‰. The elastic modulus of the laminate $E_{f}=170.4\;GPa$ was adopted from the experimental measurements (Table 2). This value is consistent with that specified in a product data sheet (170 GPa). However, the tensile strength measured experimentally and reported in Table 2 is lower than that provided in the product data sheet (the producer estimates the mean value of $f_{fu}$ to be 3100 MPa). The discrepancy is likely attributable to excessive clamping pressure in the grips of the testing machine, which damaged the outer fibres of the laminate. This resulted in a reduction of the laminate cross-section and premature rupture of the specimen near the grip area. It should additionally be noted that the ultimate strains $\varepsilon_{fu}$ measured during the beam tests (in which laminate rupture occurred) were approximately 18‰. Therefore, it can be concluded that the tensile strength of the laminate is 170.4 GPa × 18‰ = 3067 MPa, a value in close agreement with that provided by the manufacturer. Thus, for further numerical simulations, $f_{fu}=3100\;MPa$ was assumed. The stress–strain relationship for the CFRP laminate is illustrated in Figure 12a.

#### 3.2.4. Steel-to-Concrete and Laminate-to-Concrete Interfaces

The incremental constitutive relationships of the laminate-to-concrete and steel-to-concrete interface were assumed in the decoupled form:

(5) $$∆t=K∆\bar{u},\;\;\;∆t=\left[\begin{array}{c} ∆t_{ny}\\ ∆t_{nz}\\ ∆t_{t} \end{array}\right],\;\;\;∆\bar{u}=\left[\begin{array}{c} ∆{\bar{u}}_{ny}\\ ∆{\bar{u}}_{nz}\\ ∆{\bar{u}}_{t} \end{array}\right],\;\;\;K=\left[\begin{array}{ccc} K_{ny} & 0 & 0\\ 0 & K_{nz} & 0\\ 0 & 0 & K_{t} \end{array}\right],$$

where $∆t_{ny}$ and $∆t_{nz}$ are the increments of the normal tractions in the direction of the y and z axes of the interface, respectively; $∆t_{t}$ is the increment of the shear traction (along the laminate or reinforcing bar); $∆{\bar{u}}_{ny}$ and $∆{\bar{u}}_{nz}$ are the increments of the relative displacements in the normal directions of the y and z axes of the interface, respectively; $∆{\bar{u}}_{t}$ is the increment of the tangential relative displacement (slips between concrete and steel or concrete and laminate); $K_{ny}\;and\;K_{nz}$ are the stiffnesses in the normal directions of the y and z axes of the interface; and $K_{t}$ is the tangential stiffness. In the normal directions, the linear behavior of the interface was assumed with the high penalty values of 4.0·10^4^ N/mm^3^ for the stiffnesses $K_{ny}\;and\;K_{nz}$. The tangential stiffness is described by the formula


(6) $$K_{t}=\frac{{∆g}_{t.i}}{{∆\bar{u}}_{t}}\;,$$

The function $g_{t.i}=g_{t.i}\left({\bar{u}}_{t}\right),$ where i=s,l describes the physical relationships between the tangential traction (shear) and the slip in the concrete-to-steel (i=s) and concrete-to-laminate (i=l) interfaces. For reinforcing steel, the formula according to [79] was adopted:

(7) $$g_{t.s}=t_{s.max}\cdot \left(1-\exp\left[{\left(\frac{-40\cdot {\bar{u}}_{t}}{Ø}\right)}^{0.6}\right]\right),$$

where Ø is the diameter of the tensile reinforcing bar, $t_{s.max}={0.9\cdot \left(f_{c}/MPa\right)}^{2/3}\cdot MPa$, $f_{c}$ is the compressive strength of concrete, and ${\bar{u}}_{t}$ is the slip between concrete and steel. The initial stiffness $K_{t0.s}$ for the relationship in Equation (7) is calculated for traction equal to $0.02\cdot t_{s.max}$. The traction–slip behavior between the rebar and concrete is illustrated in Figure 12b.

The formula for the function $g_{t.l}$ for the laminate-to-concrete interface was taken in the form [57]

(8) $$g_{t.l}=t_{l.max}\cdot 2.5\cdot {\left(1-0.271\frac{{\bar{u}}_{t}}{{\bar{u}}_{t0}}\right)}^{2}\cdot \sin\left[\pi \left(1-0.271\frac{{\bar{u}}_{t}}{{\bar{u}}_{t0}}\right)\right]\;{\bar{u}}_{t}\leq 3.682{\bar{u}}_{t0}\;,$$

where $t_{l.max}={1.152\cdot \gamma^{0.138}\cdot \left(f_{c}/MPa\right)}^{0.613}\cdot MPa$, ${\bar{u}}_{t0}={0.201\cdot \gamma^{0.284}\cdot \left(f_{c}/MPa\right)}^{0.006}\cdot mm$, γ is the groove height-to-width ratio taken equal to 19/6, and ${\bar{u}}_{t}$ is the slip between the concrete and laminate. The initial stiffness $K_{t0.l}$ for the laminate-to-concrete interface was calculated as $K_{t0.l}=g_{t.l}\left({\bar{u}}_{el}\right)/{\bar{u}}_{el}$, where ${\bar{u}}_{el}=0.01{\bar{u}}_{t0}$. The bond–slip model for the laminate-to-concrete interface is illustrated in Figure 12c.

The interface between the concrete and the load steel plates or the support steel plate was modeled as elastic, with the stiffness in tangential directions equal to 0.1 N/mm^3^. In the normal direction, a high penalty stiffness was adopted with a value of 4.0·10^4^ N/mm^3^.

#### 3.2.5. Summary of the Adopted Material Parameters

The set of material parameters defining the constitutive models of the individual components of the beams, together with the procedure used for their determination, is summarized in Table 4.

### 3.3. Strategy of Numerical Simulations

The numerical analyses closely replicated the experimental procedure, which comprised several stages involving not only variations in loading sequences but also the progressive introduction of new structural components, namely the NSM CFRP strengthening system. Consequently, a phased analysis was required in the calculations.

#### 3.3.1. Loadings

Four loading types were included in the numerical simulations: self-weight, concrete shrinkage, prestressing of the CFRP laminates, and external loading. The self-weight was modeled as uniformly distributed body forces. The external load was modeled as a concentrated force applied at the midpoint of the load transfer beam. Shrinkage was modeled as imposed strains in the concrete, with the time-dependent evolution defined in accordance with Eurocode 2 [63]. The prestress was modeled as a concentrated force applied to the end of the laminate. The initial value of the prestress force was determined in such a way that after bonding the laminate and releasing the initial prestress force, the strains in the laminate were equal to the values measured experimentally.

#### 3.3.2. Phased Analysis

The numerical simulations were conducted in the following phases:

- Phase 1—reinforced concrete element prior to strengthening. The model components activated in this phase comprised the concrete matrix elements, the steel plates, the interface between the steel plates and the beam, the steel reinforcement, the steel-to-concrete interface, and the kinematic boundary conditions. The loads were applied incrementally in the following sequence: shrinkage, self-weight, and finally preloading (applicable only to L-type beams).
- Phase 2—prestressing. In this phase, the laminate elements and temporary laminate support constraints were additionally activated. The prestressing was incrementally applied as the concentrated force at the end of the laminate near the support.
- Phase 3—releasing of the prestressing force. In this phase, the additional laminate supports were deactivated, and the concrete-to-laminate interface elements were activated. The prestressing force was incrementally decreased to zero.
- Phase 4—loading to failure. In this phase, a new constraint in the z direction localized on the steel transfer beam was added to the model. The displacement-controlled loading procedure was applied in this phase.

In the case of passively strengthened beams (this type of beam was not experimentally tested), Phase 2 and Phase 3 were replaced with Phase 2a. In this phase, the laminate and laminate-to-concrete interface elements were activated.

#### 3.3.3. Numerical Procedure

An incremental-iterative procedure was employed to obtain the solution. The computational process was governed by increases in the external loads and prestress throughout Phases 1 to 3. In Phase 4, the force-controlled loading method was replaced with a displacement-controlled procedure. The vertical displacement of the node located at the midpoint of the transfer beam was adopted as the displacement control parameter. Equilibrium between the internal and external forces was established using the Newton–Raphson procedure [80]. This procedure was subsequently switched to the Broyden–Fletcher–Goldfarb–Shanno (BFGS) method [80] several steps prior to the ultimate load and throughout the post-critical loading steps in Phase 4. Residual force and displacement convergence criteria were used at each load increment. At each iteration, the radius of convergence was enlarged using the line-search technique [80].

### 3.4. Mesh Dependency Study

**Figure 13:**
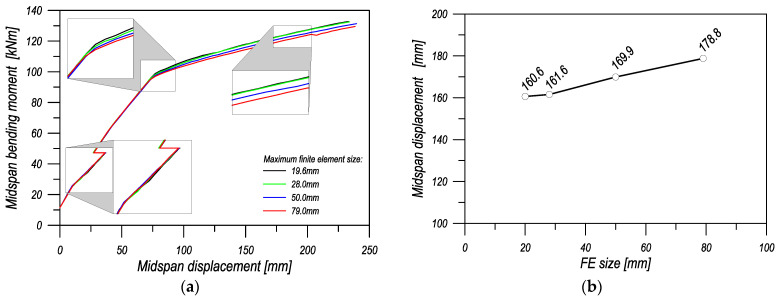
(**a**) Midspan bending moment versus midspan deflection; (**b**) midspan deflection for bending moment of 120 kNm versus finite element size.

In numerical simulations of RC structures where the cracking of concrete occurs, the mesh dependency (i.e., the dependence of the numerical solution on the finite element discretization) is crucial and, in some cases, can lead to spurious results. Therefore, in the physical model for concrete described in Section 3.2, the mesh objectivity of the finite element solution was enforced using the so-called fracture-energy regularization technique. However, excessively coarse finite element meshes may lead to an inaccurate representation of the tangent traction distribution between steel reinforcement and concrete, as well as between the laminate and the concrete. Hence, in order to assess the influence of the finite element mesh on the numerical solution, a mesh dependency study was performed. Four finite element meshes were analyzed with maximum element sizes of 19.6 mm, 28 mm (the mesh presented in Figure 6 and Figure 7), 50 mm, and 79 mm. The calculations were performed for the NSM16B-L specimen, and the results are shown in Figure 13. Figure 13a shows the relationship between the midspan bending moment and the midspan deflection for the tested meshes. Figure 13b illustrates the effect of the finite element size on the midspan deflection under a midspan bending moment of 120 kNm.

It can be concluded from Figure 13 that within the range up to steel reinforcement, the finite element mesh size does not influence the solution. This is due to the previously mentioned mesh objectification with respect to the finite element discretization for the concrete constitutive law. A minor influence appears after the yielding of the tensile reinforcing steel. These differences are presumably due to a coarse representation of the shear stress distribution along the reinforcement and the laminate in the case of the coarse finite element meshes (50 mm and 79 mm). Additionally, it should be noted that the solution stabilizes for a finite element size of 28 mm or smaller. Therefore, the mesh with a finite element size of 28 mm was selected for the numerical simulations. This mesh size ensures solution accuracy while minimising computational cost.

## 4. Results and Discussion of the Numerical Analysis

### 4.1. Load-Deformation Behavior, Cracking, and Ultimate Bending Moments

**Figure 14:**
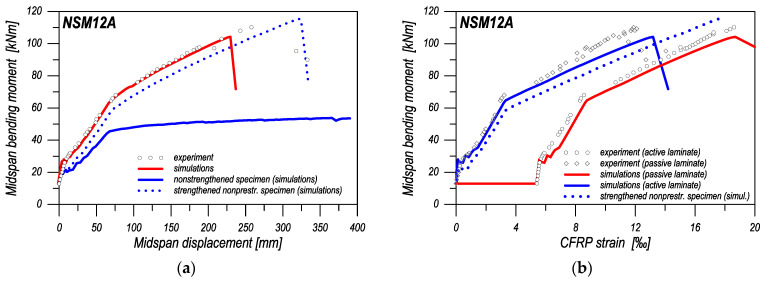
Results of simulations—beam NSM12A: (**a**) midspan bending moment versus midspan deflection; (**b**) midspan bending moment versus mean strain in the laminate.

**Figure 15:**
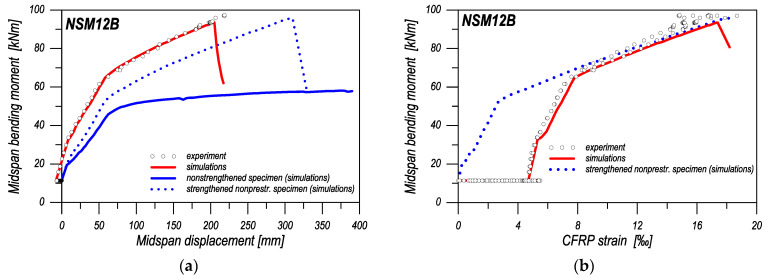
Results of simulations—beam NSM12B: (**a**) midspan bending moment versus midspan deflection; (**b**) midspan bending moment versus mean strain in the laminate.

**Figure 16:**
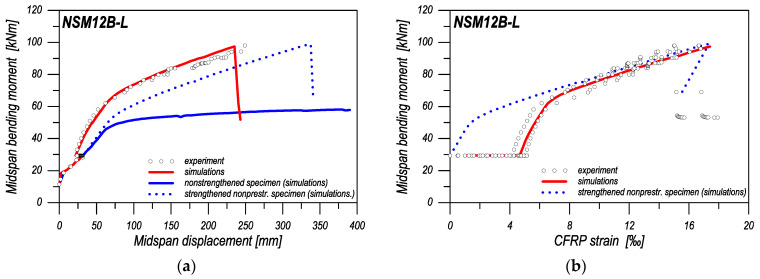
Results of simulations—beam NSM12B-L: (**a**) midspan bending moment versus midspan deflection; (**b**) midspan bending moment versus mean strain in the laminate.

**Figure 17:**
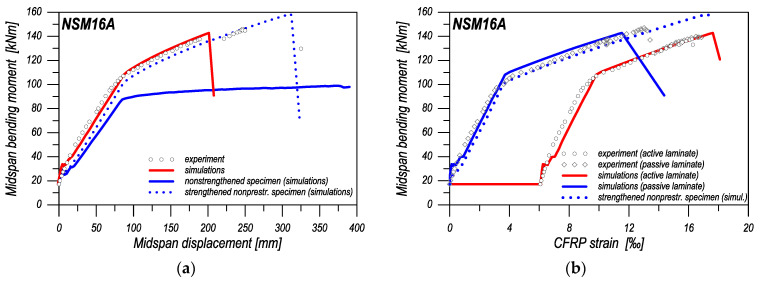
Results of simulations—beam NSM16A: (**a**) midspan bending moment versus midspan deflection; (**b**) midspan bending moment versus mean strain in the laminate.

**Figure 18:**
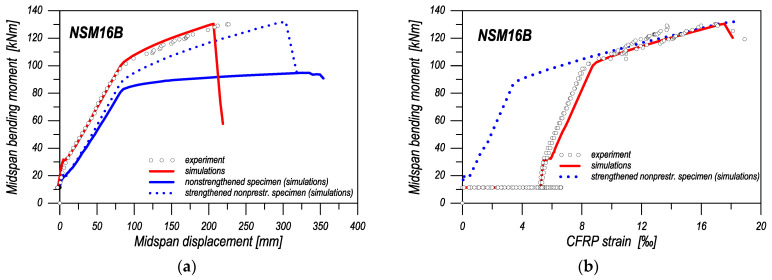
Results of simulations—beam NSM16B: (**a**) midspan bending moment versus midspan deflection; (**b**) midspan bending moment versus mean strain in the laminate.

**Figure 19:**
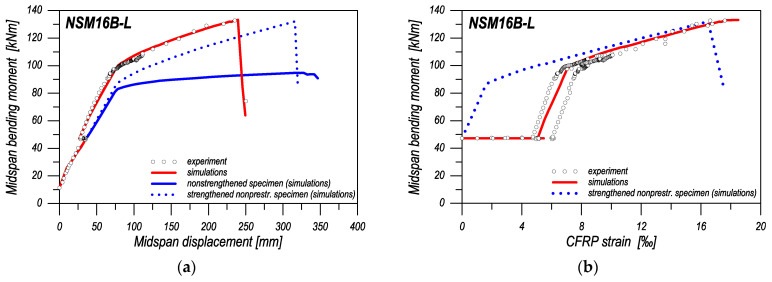
Results of simulations—beam NSM16B-L: (**a**) midspan bending moment versus midspan deflection; (**b**) midspan bending moment versus mean strain in the laminate.

The numerical simulations are compared with the experimental measurements in Figure 14, Figure 15, Figure 16, Figure 17, Figure 18 and Figure 19 and in Table 5.

Figure 14a, Figure 15a, Figure 16a, Figure 17a, Figure 18a and Figure 19a show comparisons of the vertical deflections versus the bending moments at the middle of the span of the analyzed beams. The calculated values of the deflections are in very good agreement with the deflections measured in the experiments. The numerical models correctly describe the behavior of the beams before and after strengthening. Also, the models correctly reproduce all loading stages: before and after cracking, prestressing, and after yielding of the steel reinforcement.

For specimens NSM12A, NSM12B, NSM12B-L, and NSM16A, the ultimate bending moments obtained from the calculations are slightly lower than the experimental values. This is probably caused by the numerical instability in the computations near the ultimate loads for these specimens. However, it should be emphasized that the differences between the predictions and the experimental results are minor and are limited to 1% to 6%. In both the numerical simulations and the experiments, the governing failure mechanism was rupture of the laminate in the vicinity of the crack located beneath the midspan loading point. This demonstrates that the laminate can be fully utilized without an additional end anchorage.

Slightly larger discrepancies were observed in the case of the cracking moment. The ratio between the experimentally obtained cracking moment and the calculated one ranged from 7% to 20%. One of the probable sources of these differences is the value of the concrete tensile strength $f_{t}\;$ adopted in the calculations. This value was determined based on the correlation relationships between the direct uniaxial tensile strength and the splitting tensile strength (see Table 4). A correlation coefficient of $\alpha_{sp}=1.0\;$ was assumed following the recommendation in [68]. However, the literature reports a wide range of values for this parameter, ranging from 0.7 to 1.2 [81,82]. Another potential source of discrepancy between $M_{cr.exp}$ and $M_{cr.FEM}$ is the shrinkage strain adopted in the calculations, which affected the reduction in cracking resistance. Since concrete shrinkage was not directly measured in the experiment, the values recommended in [63] were used. As indicated in [83], the actual shrinkage may deviate from the values recommended in codes of practice by up to ±30%. Considering the variability in the recommended correlation factors for concrete tensile strength, as well as the uncertainty in the actual shrinkage strain, it can be concluded that the differences between the predicted and experimental results remain within an acceptable range.

The numerical simulations confirmed the very high strengthening efficiency of the NSM CFRP technique. The increase in the load-bearing capacity of specimen NSM16 (with a higher reinforcement ratio of $\rho_{s}=0.87\%$) ranged from 37% to 43%. For specimen NSM12 ($\rho_{s}=0.49\%$), the increase in load-bearing capacity was even greater, ranging from 61% to 93%. The numerical simulations also showed that preloading and prestressing have a very limited effect on load-bearing capacity.

The increase in the cracking moment was strongly affected by the number of pretensioned CFRP strips. The increase in the cracking moment for members reinforced with two prestressed laminates ranged from 51.2% to 67%, depending on the steel reinforcement ratio of the member, with a lower reinforcement ratio corresponding to a greater influence of laminate prestressing. In the case of members with a single prestressed laminate, the increase in the cracking moment ranged from 16.7% to 32.2%, with the lowest increase observed for the member with the highest steel reinforcement ratio.

### 4.2. Strains in Laminate

In Figure 14b, Figure 15b, Figure 16b, Figure 17b, Figure 18b and Figure 19b, the strains in the CFRP laminate of experimentally tested and numerically simulated specimens are compared. For experimentally tested beams, the strains were averaged from three strain gauges located in the constant bending moment zone of the specimen. In the case of the results obtained from the numerical analysis, the laminate strains were averaged over a 300 mm length measured from the axis of symmetry. Also, in this case, the numerical simulations exhibited satisfactory agreement with the experimental observations for both the fully pretensioned specimens (B-type) and those strengthened using mixed techniques (A-type). In both scenarios, the ultimate tensile strain was attained in the CFRP laminates. Minor discrepancies were observed for elements NSM12A and NSM12B at load levels approaching the ultimate capacity. For these specimens, the largest deviations between the numerically predicted and experimentally measured failure loads were also observed. For specimens NSM12B-L and NSM16B-L, the prestress introduced experimentally into the laminates exhibited a distinct asymmetry. This asymmetry resulted from the sequential pretensioning of the CFRP laminate applied to specimens with substantially reduced stiffness. Due to the use of symmetry in the numerical models, an average level of pretension was assumed in the simulations, as shown in Figure 16b, Figure 17b, Figure 18b and Figure 19b. Despite this simplification, very good agreement between the experimental and numerical results was obtained for the global response of the structure (Table 5, Figure 16a and Figure 19a). It should also be emphasized that differences in CFRP laminate strains occurred mainly in the initial phase of loading and disappeared after the yielding of the steel reinforcement.

### 4.3. Crack Patterns

**Figure 20:**
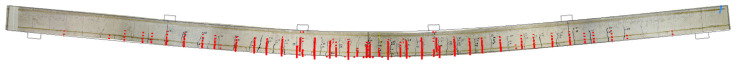
Crack patterns (localization of damages) for the ultimate load level—NSM16B, experiment versus simulation.

The presented numerical model demonstrates accurate replication of the crack patterns observed experimentally. Figure 20 shows a comparison of experimentally observed cracks and those obtained numerically for specimen NSM16B at the ultimate load. The numerical cracks are presented as red lines for the finite elements localized near the external surface of the beam. These cracks are represented by strains normal to the crack plane and are visualized as line segments localized at the integration points. Figure 20 shows the maximum normal crack strain for cases in which more than one crack develops at a given integration point.

The first fracture damage (cracks) during the numerical analysis was localized near the stirrups. Due to concrete shrinkage, tensile stress concentrations developed in the vicinity of the stirrups and main reinforcing bars. In this case, the stirrups functioned as crack initiators. Therefore, in the region of the beam where the stirrups were present, at subsequent load stages, the numerical representation of the crack patterns effectively mapped the cracks obtained experimentally. Table 6 compares the experimentally measured (at the level of the tensile reinforcement) and numerically simulated crack spacings for the constant bending moment zone of the beams. In this zone, the stirrups were not applied. Thus, crack spacing was governed by the bond–slip interaction between the tensile steel rebars and the concrete. The differences between the experimental and numerical results did not exceed 10%.

### 4.4. Active Versus Passive Strengthening

Figure 14, Figure 15, Figure 16, Figure 17, Figure 18 and Figure 19 present the results of numerical simulations for the reference specimens, i.e., those unstrengthened and those strengthened using passive techniques. These figures demonstrate that the prestressing of the CFRP laminate did not influence the ultimate load-bearing capacity of the specimen. This observation is consistent with engineering intuition and with the experimentally observed and numerically reproduced failure mechanisms involving laminate rupture accompanied by yielding of the steel reinforcement. Significant differences, however, were observed concerning the displacements at the ultimate load. For actively strengthened elements, these displacements were approximately 30% smaller, corresponding well with the initial CFRP laminate strain (from the pretensioning phase), which was equal to 33% of the rupture strain. Pretension applied to the CFRP laminate had a considerable effect on the serviceability limit state by introducing a constant reverse curvature along the strengthened zone. For specimens strengthened under self-weight only, this resulted in an increased cracking moment and reduced deflection prior to yielding. For passively strengthened specimens, the increase in the cracking moment was negligible. Also, the yielding of the steel reinforcement occurred at a higher load level compared to that in passively strengthened specimens. In the case of specimens strengthened at high external load levels (L-type, with a developed cracking pattern), the application of a pretensioned CFRP laminate led to an immediate reduction in deflection. Moreover, similarly to elements strengthened under self-weight only, prestressing reduced deflection prior to steel yielding and increased the load level required to initiate yielding of the reinforcement compared to passively strengthened specimens.

Comparing the results shown in Figure 14a, Figure 15a, Figure 16a, Figure 17a, Figure 18a and Figure 19a for the unstrengthened element and for the passively strengthened element, it can be concluded that within the load range relevant to serviceability limit states, the passive application of the laminate led to a noticeable increase in stiffness only in elements with a lower degree of steel reinforcement. For initially preloaded structures, the effect of the stiffness of a passively applied laminate was negligible.

### 4.5. Tension-Stiffening Effect for Steel Reinforcement and CFRP Laminates

**Figure 21:**
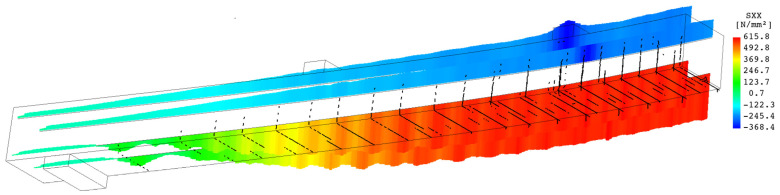
Stresses in the main (steel) reinforcement under the ultimate load—NSM16B specimen.

The numerical analysis also provided valuable information about mechanical quantities not directly measured during the experiment. Figure 21 shows the distribution of stresses in the main reinforcing steel rebars along the NSM16B specimen. These stresses are presented for the ultimate load. The stress diagrams were superimposed on the crack patterns (damage localizations) obtained from the numerical simulations. Consequently, Figure 21 clearly illustrates the tension-stiffening effect of the tensile reinforcing bars between cracks, particularly in the zone prior to yielding of the reinforcement, i.e., in the area near the support. The stress increase in the steel rebars was observed at the cracks. This stress decreased in the regions away from the cracks due to the transfer of part of the stress from the steel rebars to the concrete through the anchorage mechanism defined by the bond–slip physical law shown in Figure 12a. It is worth noting that this effect diminished in the midspan region of the specimen, where, under the ultimate load, large plastic strains developed in the steel rebars.

**Figure 22:**
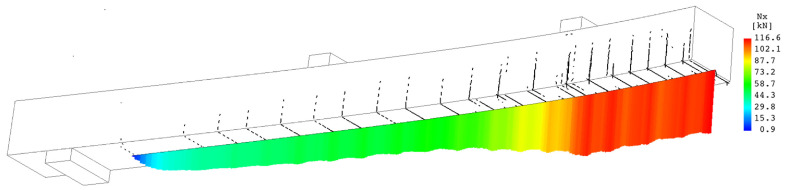
Axial tensile force in the CFRP laminate under the ultimate load—NSM16B specimen.

**Figure 23:**
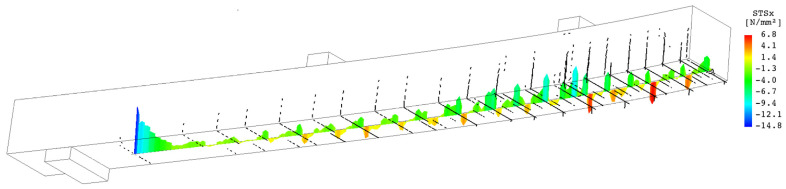
Shear stress at the concrete–CFRP interface under the ultimate load—NSM16B specimen.

An analogous mechanism to the one described above was also observed in the case of strengthening with a CFRP laminate (Figure 22 and Figure 23). Figure 22 shows the distribution of the axial tensile force along the laminate for the NSMB16-L specimen. In addition, Figure 23 shows the distribution of shear stresses (traction) between the laminate and the concrete, illustrating the anchorage effect of the laminate between cracks. The increased force in the cracked regions is clearly visible in these figures, with the gradual transfer of part of this force to the concrete between cracks. It should also be noted that the bond stresses shown in Figure 23 in the regions between cracks are much lower than the ultimate value $t_{l.max}=\;15.2\;MPa$ determined from Equation (8). Therefore, it can be concluded that the laminate does not undergo significant slips between cracks and that the strains of the strengthening system remain compatible with the concrete throughout the entire loading process, i.e., up to laminate failure—a condition that is not always achieved with other strengthening techniques such as the EB method [84].

### 4.6. Sensitivity to Assumed Material Parameters

**Figure 24:**
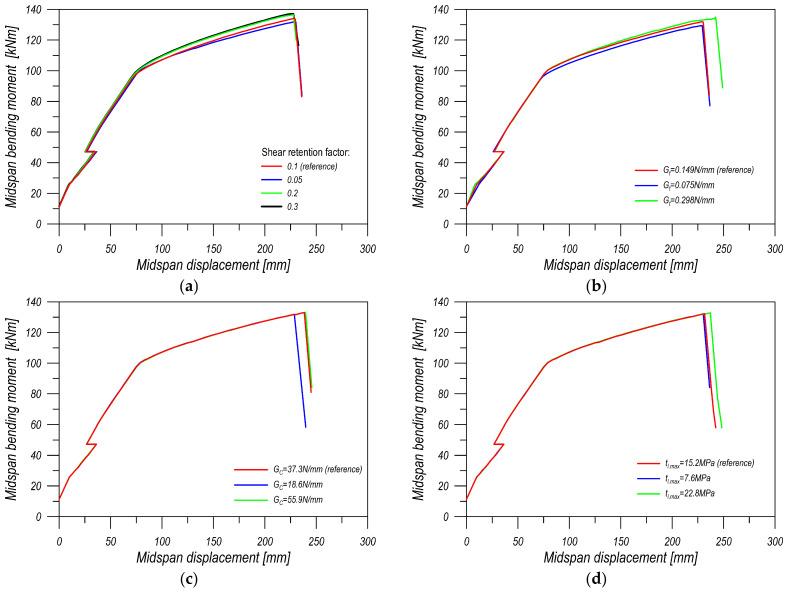
The influence of the (**a**) shear retention factor; (**b**) tensile fracture energy; (**c**) compressive fracture energy; (**d**) maximum traction at the CFRP-to-concrete interface on the load-displacement behavior of the NSM16B-L specimen.

**Figure 25:**
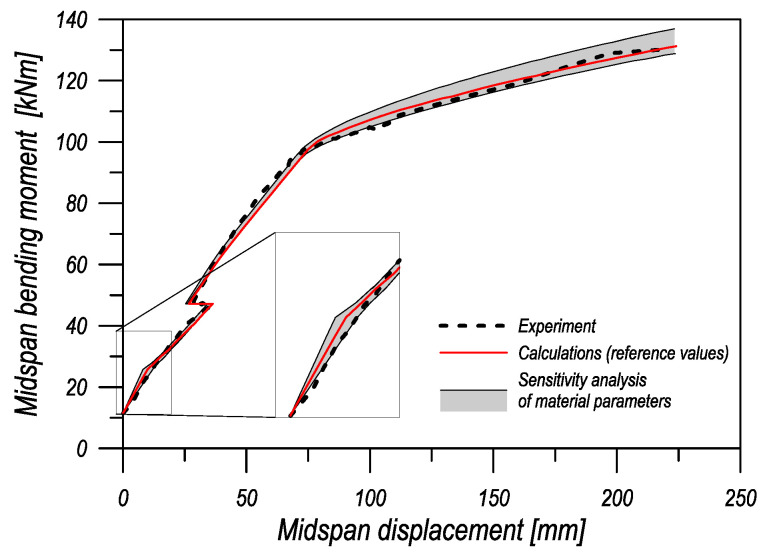
Effect of material parameters on the structural behavior of the NSM16B-L specimen.

Although the comparison between the results of the computational simulations and the experimental data demonstrates the good predictive capability of the model, the question arises as to the influence of non-trivial material parameters (i.e., parameters not obtained directly from the experiments summarized in Table 2) on the mechanical response of the structure. Therefore, a sensitivity analysis of the following model parameters was performed: the shear retention factor β, the tensile fracture energy of concrete $G_{t}$, the compressive fracture energy of concrete $G_{c}$, and the maximum traction $t_{max}$ in the bond–slip law for the CFRP–concrete interface (Figure 24 and Figure 25).

The effect of the shear retention factor is shown in Figure 24a. The figure indicates a minor influence of this material parameter on the stiffness of the model in the post-yield range of the reinforcing steel. Similarly, a minor (approximately 1%) effect on the load-carrying capacity of the simulated element was observed within the analyzed range of the shear retention factor (β=0.05 to β=0.3). It should also be noted that for β values exceeding 0.2, further changes in this parameter did not affect the structural response.

The influence of fracture energy $G_{t}$ on the structural response became apparent in the initial phase of tension, during crack initiation, and after yielding of the reinforcing steel. However, similarly to the effect of the coefficient *β*, the influence of $G_{t}$ on stiffness and load-bearing capacity was minor. The differences in the mechanical response of the model did not exceed 4% over the range of analyzed values from 0.075 N/mm to 0.298 N/mm (Figure 24b).

In the case of the compressive fracture energy and the maximum shear stress (traction) in the CFRP-to-concrete bond–slip interface, no stiffness-related effect was observed (compare Figure 24c,d for $G_{c}$ and $t_{l.max}$, respectively). A slight difference was recorded only with regard to the ultimate load, amounting to 1% for extreme values of the analyzed parameters.

In order to show the sensitivity of the analyzed parameters on the global load-deformation behavior of the beams, the variations in the numerical results for all analyzed parameters are shown in Figure 25. The shaded region in this figure represents the influence of the variability of the analyzed model parameters on the mechanical response of the structure. The range of the discrepancies between the results of the numerical simulations was relatively narrow. The largest differences were observed in the initial stage of loading, i.e., during the formation of the stabilized crack phase and after the yielding of the steel reinforcement. It should also be noted that the numerical solution obtained for the reference parameters (according to Table 4) correctly reproduced the behavior of the tested structural element in both loading phases.

Summarizing the results presented above, it can be concluded that the numerical study demonstrates the high predictive capability of the model and accurately captures the flexural response of beams strengthened with CFRP laminates using the near-surface-mounted technique. However, it should be emphasized that the proposed computational approach is limited to the analysis of elements for which the anticipated failure mode is governed by the tensile mechanism (i.e., yielding of the reinforcement and rupture of the laminate at a crack) and does not require strengthening for other failure mechanisms, for example, shear failure. Despite this limitation, the proposed approach can be applied in practice, especially in expert-oriented design problems where simplified methods (i.e., analytical methods based on cross-sectional analysis) are not pertinent and only more sophisticated nonlinear numerical simulations must be applied. Also, this approach can be employed as a research tool.

## 5. Summary and Conclusions

This paper presents a numerical approach to modeling the strengthening procedure of flexural elements using pretensioned CFRP laminates embedded in the concrete cover. A particular emphasis was placed on the realistic representation of the strengthening process, understood as the application of a strengthening system to preloaded and damaged (cracked) structural elements, i.e., structures that cannot be fully unloaded prior to the application of the strengthening system. The methodology for modeling such a strengthening process was concisely and comprehensively described in this paper. The numerical results were compared with experimental data. Additionally, simulations were performed for both unstrengthened elements and passively strengthened elements.

The following conclusions can be drawn from the comparative analysis of the experimental and numerical results:

- Good agreement was observed between the experimental measurements and numerical simulations. The cracking mechanism of the elements prior to strengthening, induced by preloading, followed by the strengthening stage and the failure of the strengthened elements, was correctly reproduced by the numerical model.
- The comparison of beams strengthened under self-weight and under external preloading revealed that the load-bearing capacity was independent of the preloading level.
- The experimental data and the following numerical calculations confirmed the high efficiency of strengthening flexural elements using NSM CFRP laminates. Increases in the load-carrying capacity of 37% to 93% were observed for the strengthened elements compared with the unstrengthened reference (control) specimen.
- A comparable increase in load-bearing capacity was observed for passively strengthened specimens. It must be emphasized, however, that at the ultimate load, the deformations of passively strengthened members were approximately 30% greater than those of actively strengthened specimens. The principal advantage of active strengthening over passive solutions lies in the substantial reduction of deformations in actively strengthened structures.
- In both the numerical simulations and the experimental program, the governing failure mechanism was rupture of the laminate. This demonstrates that in the NSM CFRP strengthening technique, the laminate can be fully utilized without an additional end anchorage.
- The proposed numerical model correctly reproduced the experimentally obtained crack patterns and the cracking moment.
- The number of prestressed CFRP laminates significantly influenced the increase in the cracking moment. The numerical analysis demonstrated that the application of a single prestressed laminate strip resulted in increases in the cracking moment ranging from 16.7% to 32.2%, whereas the application of two prestressed laminate strips yielded increases ranging from 51.2% to 67%.
- Numerical simulations showed that strengthening with a passive CFRP laminate did not affect the cracking moment.
- The tension-stiffening effect of the steel reinforcement was successfully replicated in the numerical study. An analogous effect was further exhibited by the CFRP laminate, both for elements strengthened under self-weight and for those strengthened under external preloading.
- It was demonstrated that the traction between the CFRP laminate and the concrete did not exceed the maximum value, even at the ultimate load. Thus, the relative slips of the laminate between cracks were limited. Therefore, the strains of the strengthening system remained compatible with the concrete throughout the entire loading process, i.e., up to the rupture of the laminate.
- The sensitivity analysis of the non-obvious material model parameters—i.e., those not directly obtained from experimental measurements—demonstrated that the recommendations from the literature summarized in Table 4 yielded an accurate description of the behavior of beams strengthened using the NSM technique with prestressed CFRP laminates.
- The proposed computational model can be effectively applied to predict the effects of strengthening as well as to analyze crack propagation in elements that are lightly or heavily loaded prior to strengthening. It should be noted, however, that the applicability of the proposed computational approach is limited to members for which the anticipated failure mode is governed by tensile mechanisms. Despite this limitation, the proposed approach can be applied in practical engineering situations—particularly in expert-oriented design—and also serves as a research tool in cases where simplified analytical approaches are insufficient and more sophisticated nonlinear modeling is required.

## Figures and Tables

**Table 1:** Description of tested beams, investigated parameters, and strengthening configurations.

Specimen	Tensile Steel Bar	Number of CFRP Strips		*M_u_*_0_ [kNm]	*M_p_* [kNm]	*M_p_*/*M_u_*_0_ [%]
		Passive	Active			
NSM12A	4ϕ12	2	1	46.5	13.5	25
NSM16A	4ϕ16	2	1	84.7	15.0	14
NSM12B	4ϕ12	-	2	46.5	13.5	25
NSM16B	4ϕ16	-	2	84.7	15.0	14
NSM12B-L	4ϕ12	-	2	46.5	27.9	60
NSM16B-L	4ϕ16	-	2	84.7	50.9	60

*M_u_*_0_—design moment-bearing capacity of the unstrengthened specimen estimated based on the cross-sectional analysis [60]; *M_p_*—preloading bending moment; *M_p_*/*M_u_*_0_—preloading ratio.

**Table 2:** Strength characteristics of structural materials.

Material	Property	Unit	Series A				Series B			
			NSM12		NSM16		NSM12		NSM16	
			8	12	8	16	8	12	8	16
Steel	*E_s_*	[GPa]	186.1	191.3	196.5	198.0	205.5	214.0	205.5	204.9
	*f_y_*	[MPa]	416.2	539.6	555.8	595.0	554.9	563.4	554.9	578.3
	*f_u_*	[MPa]	734.1	627.5	646.0	672.0	608.9	651.7	608.9	693.8
Concrete	*f_c_*	[MPa]	46.0		53.9		51.0		52.0	
	*f_c,cube_*	[MPa]	44.9		59.5		60.0		60.1	
	*f_ct,sp_*	[MPa]	3.95		4.30		4.5		4.1	
	*E_cm_*	[GPa]	25.3		24.0		25.8		24.3	
CFRP	*E_f_*	[GPa]	170.4							
	*f_fu_*	[MPa]	2551							
	*ε_fu_*	[‰]	13.6							

**Table 3:** Summary of experimental results.

	NSM12A	NSM12B	NSM12B-L	NSM16A	NSM16B	NSM16B-L
Failure mod.	R	R	R	R + CC	R	R
*M*_p_/*M*_u0_ [%]	25	25	60	14	14	60
*M_cr_* [kNm]	22.0	26.0	-	27.3	29.0	-
*M_u_* [kNm]	110.2	97.0	98.0	146.9	130.0	133.0
*ε_fp_* [‰]	5.4	4.6/4.7	4.3/5.1	6.1	5.4/5.4	5.2/6.1
*v_p_* [mm]	−1.9	−6.4	−7.7	−1.7	−3.6	−5.2
*v_Mu_* [mm]	258	248	249	245	227	235

R—rupture of CFRP; CC—concrete crushing; *M_p_*—preloading bending moment; *M_u_*—ultimate bending moment; *M_cr_*—cracking moment; *M_u_*_0_—ultimate bending moment of the unstrengthened specimen estimated based on the cross-sectional analysis; *ε_fp_*—strain of the CFRP laminate after prestressing; *v_p_*—negative (reverse) displacement after prestressing; *v_Mu_*—displacement at the ultimate bending moment.

**Table 4:** Summary of material parameters adopted in the numerical study.

	Experimentally Determined Parameters (Values According to Table 2)	Parameters Based on the Literature
Concrete	- compressive strength $f_{c}$; - tensile strength ${f_{t}=\alpha_{sp}f}_{ct.sp},\;\alpha_{sp}=1.0$ [68]; - elastic modulus $E_{c}=E_{cm}$	- shrinkage strain $\varepsilon_{cs}\left(t\right)$ [63]; - tensile stress–strain relationship $\sigma_{t}=\sigma_{t}\left(\varepsilon \right)$ [65]; parameters $c_{1}$, $c_{2}$ [65]; - tensile fracture energy $G_{t}$ ^(a)^ [68]; - Poisson’s ratio [63]; - shear retention factor β [69,70,71,72]; - compressive stress–strain relationship $\sigma_{c}=\sigma_{c}\left(\varepsilon \right)$ [73]; - strain at compressive strength $\varepsilon_{c1}$ [73]; - compressive fracture energy $G_{c}$ ^(a)^ [77]
Steel	- yield stress $f_{y}$; - tensile strength $f_{u}$; - elastic modulus $E_{s}$	-
CFRP	- elastic modulus $E_{f}$	- tensile strength of laminate $f_{fu}$ ^(b)^; - strain for tensile strength $\varepsilon_{fu}$ ^(b)^
Concrete-to-steel bond–slip law	-	- bond–slip relationship $g_{t.s}=g_{t.s}\left({\bar{u}}_{t}\right)$ [79]; - maximum traction (shear) $t_{s.max}$ ^(a)^ [79]
Concrete-to-laminate bond–slip law	-	- bond–slip relationship $g_{t.l}=g_{t.l}\left({\bar{u}}_{t}\right)$ [57]; - maximum traction (shear) $t_{l.max}$ ^(a)^ [57]; - slip for the maximum traction ${\bar{u}}_{t0}$ ^(a)^ [57]

^(a)^ value correlated with experimental compressive strength $f_{c}$; ^(b)^ data provided by the manufacturer.

**Table 5:** Results comparison: experimental versus calculated.

Element	$M_{u.exp}$	$M_{u.FEM}$	$\frac{M_{u.exp}}{M_{u.FEM}}$	$M_{u0.FEM}$ [kNm]	$\eta_{u}$ [%]	$M_{cr.exp}$	$M_{cr.FEM}$	$M_{cr0.FEM}$	$\frac{M_{cr.exp}}{M_{cr.FEM}}$	$\eta_{cr}$
	[kNm]	[kNm]				[kNm]	[kNm]	[kNm]		[%]
NSM12A	110.2	104.3	1.06	54.0	93.1	22.0	25.8	19.5	0.85	32.3
NSM12B	97.0	93.5	1.03	58.1	60.9	26.0	32.4	19.4	0.80	67.0
NSM12B-L	98.0	97.5	1.01	58.1	67.8	-	-	-	-	-
NSM16A	146.9	142.8	1.03	99.6	43.4	27.3	33.5	28.7	0.81	16.7
NSM16B	130.0	130.4	1.00	94.9	37.4	29.0	31.0	20.5	0.93	51.2
NSM16B-L	133.0	133.2	1.00	94.9	40.4	-	-	-	-	-

$M_{u.exp}$—experimental ultimate bending moment; $M_{u.FEM}$—ultimate bending moment from numerical simulations; $M_{u.0.FEM}$—calculated ultimate bending moment of the reference (non-strengthened) specimen; $\eta_{u}=\left(M_{u.FEM}-M_{u0.FEM}\right)/M_{u0.FEM}$—strengthening effectiveness; $M_{cr.exp}$—experimentally determined cracking moment; $M_{cr.FEM}$—calculated cracking moment for strengthened specimens; $M_{cr0.FEM}$—calculated cracking moment for the reference (non-strengthened) specimens; $\eta_{cr}=\left(M_{cr.FEM}-M_{cr0.FEM}\right)/M_{cr0.FEM}$.

**Table 6:** Mean crack spacing within the constant moment zone: experiment versus simulations.

	NSM12A	NSM12B	NSM12B-L	NSM16A	NSM16B	NSM16B-L
$s_{rm.exp}$ [mm]	93.2	108.0	103.3	90.1	87.2	103.9
$s_{rm.FEM}$ [mm]	90.4	89.5	110.0	90.4	90.4	105.4
$\frac{s_{rm.exp}}{s_{rm.FEM}}$	1.03	1.10	0.94	0.99	0.97	0.99

$s_{rm.exp}$—the experimentally determined mean crack spacing; $s_{rm.FEM}$—the mean crack spacing obtained in the numerical simulations.

## Data Availability

The original contributions presented in this study are included in the article. Further inquiries can be directed to the corresponding author.
